# Web-Based Asynchronous Tool to Facilitate Communication Between Primary Care Providers and Cancer Specialists: Pragmatic Randomized Controlled Trial

**DOI:** 10.2196/40725

**Published:** 2023-01-18

**Authors:** Bojana Petrovic, Jim A Julian, Clare Liddy, Amir Afkham, Sharon F McGee, Scott C Morgan, Roanne Segal, Jonathan Sussman, Gregory R Pond, Mary Ann O'Brien, Jacqueline L Bender, Eva Grunfeld

**Affiliations:** 1 Department of Family and Community Medicine University of Toronto Toronto, ON Canada; 2 Dalla Lana School of Public Health, University of Toronto Toronto, ON Canada; 3 Department of Oncology, McMaster University Hamilton, ON Canada; 4 Bruyère Research Institute Ottawa, ON Canada; 5 Department of Family Medicine, University of Ottawa Ottawa, ON Canada; 6 Ontario Health East Ottawa, ON Canada; 7 The Ottawa Hospital Cancer Centre Ottawa, ON Canada; 8 Department of Radiology, Radiation Oncology and Medical Physics, Faculty of Medicine, University of Ottawa Ottawa, ON Canada; 9 Cancer Rehabilitation and Survivorship, Department of Supportive Care, Princess Margaret Cancer Centre Toronto, ON Canada; 10 Institute of Health Policy, Management and Evaluation, University of Toronto Toronto, ON Canada; 11 Ontario Institute for Cancer Research Toronto, ON Canada

**Keywords:** electronic communication, coordination of care, cancer, primary care

## Abstract

**Background:**

Cancer poses a significant global health burden. With advances in screening and treatment, there are now a growing number of cancer survivors with complex needs, requiring the involvement of multiple health care providers. Previous studies have identified problems related to communication and care coordination between primary care providers (PCPs) and cancer specialists.

**Objective:**

This study aimed to examine whether a web- and text-based asynchronous system (eOncoNote) could facilitate communication between PCPs and cancer specialists (oncologists and oncology nurses) to improve patient-reported continuity of care among patients receiving treatment or posttreatment survivorship care.

**Methods:**

In this pragmatic randomized controlled trial, a total of 173 patients were randomly assigned to either the intervention group (eOncoNote plus usual methods of communication between PCPs and cancer specialists) or a control group (usual communication only), including 104 (60.1%) patients in the survivorship phase (breast and colorectal cancer) and 69 (39.9%) patients in the treatment phase (breast and prostate cancer). The primary outcome was patient-reported team and cross-boundary continuity (Nijmegen Continuity Questionnaire). Secondary outcome measures included the Generalized Anxiety Disorder Screener (GAD-7), Patient Health Questionnaire on Major Depression, and Picker Patient Experience Questionnaire. Patients completed the questionnaires at baseline and at 2 points following randomization. Patients in the treatment phase completed follow-up questionnaires at 1 month and at either 4 months (patients with prostate cancer) or 6 months following randomization (patients with breast cancer). Patients in the survivorship phase completed follow-up questionnaires at 6 months and at 12 months following randomization.

**Results:**

The results did not show an intervention effect on the primary outcome of team and cross-boundary continuity of care or on the secondary outcomes of depression and patient experience with their health care. However, there was an intervention effect on anxiety. In the treatment phase, there was a statistically significant difference in the change score from baseline to the 1-month follow-up for GAD-7 (mean difference −2.3; *P*=.03). In the survivorship phase, there was a statistically significant difference in the change score for GAD-7 between baseline and the 6-month follow-up (mean difference −1.7; *P*=.03) and between baseline and the 12-month follow-up (mean difference −2.4; *P*=.004).

**Conclusions:**

PCPs’ and cancer specialists’ access to eOncoNote is not significantly associated with patient-reported continuity of care. However, PCPs’ and cancer specialists’ access to the eOncoNote intervention may be a factor in reducing patient anxiety.

**Trial Registration:**

ClinicalTrials.gov NCT03333785; https://clinicaltrials.gov/ct2/show/NCT03333785

## Introduction

### Background

Cancer poses a significant global health burden [1]. The complexity of patients’ needs is increasing owing to a growing and aging population and the consequences of undergoing complex cancer treatment [2]. Patients with cancer can have high needs because of the effects of cancer itself (eg, pain), side effects of cancer treatment, and associated comorbid conditions (eg, diabetes) [3].

### Coordination and Continuity of Care

Throughout their cancer journey, patients interact with surgical, radiation, and medical oncologists; oncology nurses; and primary care providers (PCPs), among other health care professionals [4]. PCPs play an important role in cancer care, from promoting cancer screening and diagnosis to providing care during cancer treatment and follow-up [5]. Although coordination between PCPs and cancer specialists is essential to patient-centered care, research shows persistent problems related to communication, coordination, and continuity of care [6,7]. Coordination of care is defined as “the deliberate organization of patient care activities between two or more participants (including the patient) involved in a patient's care to facilitate the appropriate delivery of healthcare services” [8], whereas continuity of care refers to the patient’s perception of coherent and connected care that addresses their circumstances [9]. Although these terms have been used interchangeably in the literature, the definitions provided imply that coordination is an integral part of continuity of care [10].

### Previous Research

Previous research has demonstrated that inadequate coordination of care is related to poor communication between health care providers, which may occur because of delays in medical transcription, physicians (especially PCPs) not being copied on all patient reports, and incompatible electronic medical records (EMRs) software, making access to patient information a time-consuming and frustrating process [11]. In addition, health care providers have reported challenges related to a lack of defined and communicated roles among health care providers, especially during cancer follow-up (eg, which physician is responsible for ordering tests and addressing concerns regarding comorbidities) [12]. These communication problems can result in duplication of services [13], unnecessary appointments [11], and increased costs [14]. Poor coordination negatively affects patient experience, causing anxiety and confusion about who is in charge of their care and whom to contact regarding cancer-related questions [7].

### Electronic Communication Tools

The use of electronic communication tools to facilitate communication among providers can be helpful for patients with complex and chronic conditions who may be receiving care from multiple health care providers and in situations where patients are transferred between different health care sectors [15]. In a 2021 survey conducted by Canada Health Infoway and the Canadian Medical Association, physicians reported that they used electronic communication primarily to access laboratory tests and diagnostic results and for receipt of a hospital visit and discharge information. Within the province of Ontario (where this study was conducted), 32% of physicians reported using electronic consultation to seek or provide advice from other physicians [16]. Although previous tools have been piloted to support clinical communication [17], electronic consultation has gradually grown to include over 90 specialty services [18]. This study aimed to assess whether a web- and text-based asynchronous communication system can be used effectively to facilitate communication between PCPs and cancer specialists and improve continuity of care.

### eOncoNote Study

This study is part of a broader research program called the Canadian Team to Improve Community-Based Cancer Care along the Continuum (CanIMPACT). After conducting descriptive mixed methods research that pointed to gaps in care coordination, our team hosted a consultative workshop with multidisciplinary stakeholders, who recommended testing whether electronic consultation could help facilitate care coordination for patients with cancer [19]. The web-based communication system that we examined in this trial is a cancer-specific adaptation of Champlain Building Access to Specialists through eConsultation (BASE) eConsult [20] called “eOncoNote.” It was developed for the purpose of this study. BASE eConsult is a web-based secure communication system developed in the Champlain region (the easternmost part of Ontario, Canada, that includes the Ottawa area), which allows PCPs to access specialist advice regarding referral of a patient and potentially avoid referral and long wait times for patients to access specialist care. Previous research has demonstrated that in 43% of cases a referral to a specialist was contemplated but no longer needed after using BASE eConsult [21]. BASE eConsult has been available since 2010 [20], and >50% of PCPs in the Champlain region are registered users [18]. BASE eConsult and eOncoNote, based on the same platform, have a similar process of sending messages between a PCP and a specialist. However, in the BASE eConsult system, communication is initiated by a PCP [22] and the specialist is the recipient [23], whereas in eOncoNote, communication was initiated by a cancer specialist.

### Research Questions

The primary research question was “Does PCP and cancer specialist access to eOncoNote affect patient-reported continuity of care?” The secondary research questions were as follows: (1) “Does PCP and cancer specialist access to eOncoNote affect other patient-reported outcomes including anxiety, depression, and patient experience with health care?” and (2) “How does access to eOncoNote influence communication between PCPs and cancer specialists?”

## Methods

### Design and Intervention

We conducted a pragmatic randomized controlled trial [24] that focused on 3 malignancies: localized breast and prostate cancer for patients during their treatment phase and breast and colorectal cancer for patients who were in the survivorship phase and were transitioning back to primary care. These disease sites were selected as they are among the highest incidence and prevalence cancers [25,26].

**Figure 1:**
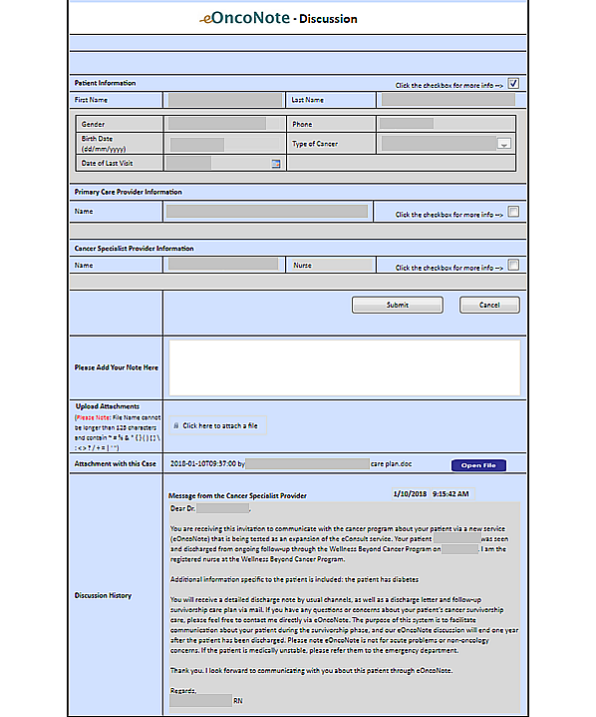
Screenshot of eOncoNote discussion page.

**Figure 2:**
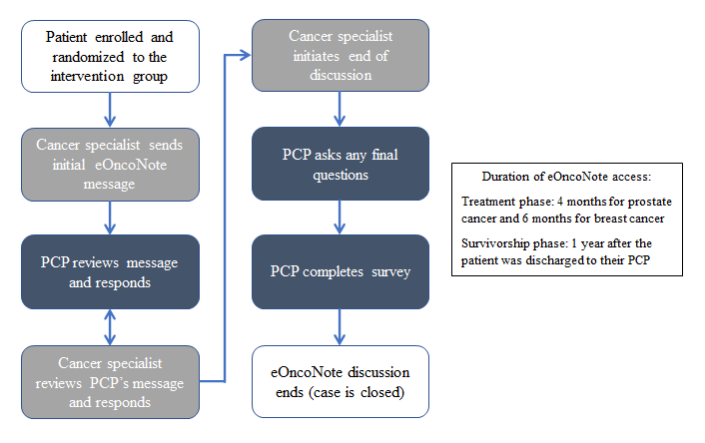
eOncoNote intervention process flowchart. PCP: primary care provider.

For the intervention group, PCPs and cancer specialists used eOncoNote in addition to the usual methods of communication (eg, visit notes, telephone, and fax). At the time of providing consent, patients were informed that the intervention involved communication between their PCP and cancer specialist via eOncoNote. After a patient provided consent to participate and was randomized to the intervention group, the cancer specialist logged onto the eOncoNote website and sent the PCP an invitation to communicate (refer to Figure 1 for the screenshot). Each time a message was sent, the recipient received an email notification prompting them to log into the eOncoNote website to view and respond to the message (refer to Figure 2 for the eOncoNote process). If a PCP did not respond to the invitation to communicate, the research assistant (RA) contacted them up to 3 times to remind them to respond to the cancer specialist. Similarly, the RA contacted them up to 3 times to remind them to close the case discussion.

Access to the website, which was available free of charge, was only provided to the cancer specialists and PCPs whose patients had consented to participate in the study. Cancer specialists and PCPs were able to contact the RA and the BASE eConsult team if they required technical support. Each notification email included contact information for support and a quick start guide with step-by-step instructions and screenshots as a reminder about the eOncoNote communication process. For the control group, PCPs and cancer specialists used the usual methods of communication only.

### Participants

Two groups of patients were recruited: (1) patients receiving active cancer treatment (ie, those who were receiving care from a medical oncologist for early-stage breast cancer or from a radiation oncologist for localized prostate cancer) and (2) patients who transitioned to survivorship care (ie, those who have completed treatment for breast or colorectal cancer and were being discharged to their PCP). The treatment and survivorship phases of cancer care were selected to examine the patients’ experiences as cancer survivors (using the National Coalition for Cancer Survivorship’s definition from the time of diagnosis onward [27]). We recruited participants separately in these 2 phases because patients interact with various health care providers as they move along the cancer care continuum. Patient inclusion and exclusion criteria are described in Textbox 1.

**Inclusion and exclusion criteria.**

**Inclusion criteria**

- At least 18 years old
- No prior history of cancer in the past 5 years (those with nonmelanoma skin cancer could participate)
- Receiving adjuvant chemotherapy for early-stage breast cancer, radical or adjuvant radiation therapy for localized prostate cancer, or completed adjuvant therapy for breast or colorectal cancer, with the intent of being discharged for survivorship follow-up care to their primary care provider (PCP)

**Exclusion criteria**

- Participating in another study requiring ongoing questionnaire completion
- Does not have a PCP
- Their PCP has another patient enrolled in the trial
- Unable to read and write in English
- Unable to provide informed consent

PCPs were eligible to participate if they were a licensed family physician or nurse practitioner, their patient consented to be enrolled in the study (and they did not have any other patients enrolled), and they were previously registered on Champlain BASE eConsult. Cancer specialists included medical oncologists and radiation oncologists (for the treatment phase) and nurses from the survivorship program (survivorship phase).

### Procedures

Patients were recruited from a large academic hospital cancer center in Ottawa, Ontario, Canada. This site was selected because it was located in a region where PCPs had access to the eConsult platform since 2010 [20]. Potential participants who met the inclusion criteria were identified by the cancer specialists and invited to meet the study RA. Following informed consent, the participants completed the baseline questionnaire, and the RA registered them in the trial. The participants were randomized in a 1:1 ratio to the intervention or control group using the web-based randomization system of the Ontario Clinical Oncology Group [28]. The random allocation was a computer-generated sequence, with a block size of 4. Patients completed the first follow-up (FUP1) questionnaires at 1 month (treatment phase) or 6 months (survivorship phase) following randomization. They completed the second follow-up (FUP2) questionnaires at 4 months or 6 months following randomization for patients receiving treatment for prostate or breast cancer, respectively, and 12 months following randomization for patients in the survivorship phase. All patients had the option to complete follow-up questionnaires by telephone or on the web using Qualtrics survey software [29].

### Measures

#### Patient Questionnaires

Continuity of care, the primary outcome, was assessed using the Nijmegen Continuity Questionnaire (NCQ) team and cross-boundary continuity (TCC) subscale. TCC is defined as a patient’s perception of the communication of pertinent patient information and cooperation between providers from different settings, ensuring that care is connected [30]. The TCC subscale consists of 4 items (eg, “these care providers pass on information to each other very well”), which are scored on a 5-point Likert scale ranging from “strongly disagree” to “strongly agree,” with an additional response option “I do not know.” A higher score on the NCQ reflects increased patient-perceived continuity of care [31]. The NCQ has shown internal consistency ranging from α=.86 to .96. As the measure was not specific to cancer, participants were instructed to answer the items about their interaction with their cancer specialist (medical oncologist or radiation oncologist for patients in the treatment phase for breast or prostate cancer, respectively, or oncology nurse for patients in the survivorship phase).

Secondary outcome measures included the Generalized Anxiety Disorder Screener (GAD-7) [32], Patient Health Questionnaire on Major Depression (PHQ-9) [33], and Picker Patient Experience Questionnaire (PPE-15) [34]. The GAD-7 [32] is a 7-item measure used to screen for generalized anxiety, whereas the PHQ-9 [33] is a 9-item measure of depression, both of which have been validated in primary care settings. The PPE-15 asks about various aspects of a patient’s health care experience (eg, respect for patient preferences) [34]. The GAD-7, PHQ-9, and PPE-15 have demonstrated high internal consistency (α=.80 to .89) [32-34]. A higher score for each of these 3 measures indicates greater anxiety, greater depression, and more problems associated with patient health care experience.

In addition to the outcome measures, patients completed sociodemographic questions (eg, racial or cultural group, marital status, and education) [35] and questions assessing multimorbidity [36] at baseline. At each assessment (baseline, FUP1, and FUP2), patients also completed questions regarding their health care use in the past month (eg, when they last saw their PCP, the reason for their last PCP visit, and other health care providers with whom they had contact).

#### Hospital EMR Data

Data from the hospital EMR were abstracted to examine any differences between the intervention and control groups regarding the frequency of calls and faxes from PCPs and patients and the reason for the calls or faxes to the cancer center.

### Statistical Analysis

#### Overview

Statistical analyses were conducted using SPSS software (version 28.0; IBM Corp) [37] and were completed separately for patients in the survivorship and treatment phases. The primary analysis used the General Linear Model (GLM) procedure using a patient’s NCQ TCC change score from baseline-to-FUP2. The model was adjusted for the baseline scores for the NCQ subscales, including TCC, personal continuity relating to the PCP (ie, PCP knows me and shows commitment), and personal continuity relating to the cancer specialist (ie, cancer specialist knows me and shows commitment), as well as the group to which the patient was randomized (intervention or control). Additional baseline covariates in the regression model included age, sex, cancer disease site, and the number of comorbid conditions. Similar analyses were conducted for the secondary outcome measures (GAD-7, PHQ-9, and PPE-15). The primary analysis for NCQ TCC involved recoding “I do not know” responses into “neutral” owing to missing data, and a sensitivity analysis was conducted using the mean of at least half of the TCC subscale responses (eg, patients had to have answered at least two of the 4 items in the TCC subscale to be included in the analysis). This approach was selected because previous research reported high internal consistency for this subscale (α=.95 to .96) [31]. Secondary analysis was conducted using the outcome measures at FUP1. Missed items were addressed according to the recommendations for each outcome measure (except for NCQ TCC, which involved recoding). Descriptive statistics were used to summarize continuous and categorical measures, and 2-tailed *t* tests were used to examine the change in scores from baseline to FUP1 and baseline to FUP2. *P* values of .05 were considered statistically significant. Data from patients who were withdrawn from the study were not included in the analyses.

#### Sample Size Estimation

**Figure 3:**
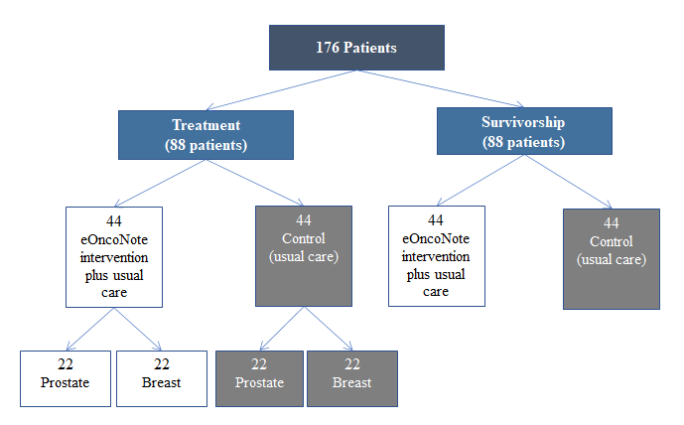
eOncoNote trial schema.

The sample size was calculated to compare 2 independent sample means and detect a difference of 0.5 in the NCQ TCC score (SD 0.75), with 80% power and a 2-sided α of 5%. The minimum number of patients required per group was 37. Considering the potential loss to follow-up, the aim was to recruit an additional 7 patients, with a recruitment target of 44 patients per group or 88 for each of the treatment and survivorship phases (176 patients in total; Figure 3). Patients in the treatment phase were stratified by disease site because different cancer specialists were involved in communicating with PCPs in this phase (ie, medical oncologists were involved for patients with breast cancer, whereas radiation oncologists were involved for those with prostate cancer).

### Ethics Approval

The study was approved by the Ottawa Health Science Network Research Ethics Board (#20170381-01H) and the Health Sciences Research Ethics Board at the University of Toronto (#34641). Patient recruitment began in February 2018 and ended in March 2020, and follow-up data collection ended in August 2020.

## Results

### Participant Characteristics

Of the 515 patients who were assessed for eligibility, 342 (66.4%) were excluded and 13 (2.5%) were withdrawn (refer to the CONSORT [Consolidated Standards of Reporting Trials] diagram in Multimedia Appendix 1). The major reasons for exclusion were that the patient’s PCP was not previously registered on Champlain BASE eConsult (n=95 for treatment; n=69 for survivorship) or the patient’s PCP already had another patient participating in the trial (n=23 for treatment; n=13 for survivorship). Although we aimed to recruit 88 patients per phase, we recruited more patients in the survivorship phase than in the treatment phase. Our original eligibility criteria included patients whose PCPs were not already registered for eConsult; however, contacting and registering PCPs for this service proved to be challenging. Thus, soon after starting the trial, the eligibility criterion was modified to include only patients whose PCPs were already registered on eConsult to avoid any burden and disappointment for patients because they could not participate in the trial if their PCP declined to register. As a result of the change to the eligibility criteria, we increased the target recruitment for the survivorship phase to 104 to compensate for the patients who had been withdrawn from the study because their PCP declined eConsult registration.

Table 1 presents the baseline and clinical characteristics by phase of cancer care and group. In the treatment phase, there were 33 patients in the intervention group and 32 patients in the control group (2 patients were withdrawn). Over half (37/65, 57%) of the patients in the treatment phase were male, and the mean age at enrollment was similar between the study groups (approximately 64, SD 11 years). Patients in the treatment phase intervention group had more comorbid conditions than those in the control group (mean 3 vs 2). In the survivorship phase, there were 43 patients in the intervention group and 52 patients in the control group. There were more patients in the control group, as 9 patients were withdrawn from the intervention group because their PCP declined to participate. Most (88/95, 93%) patients in the survivorship phase were female and breast cancer survivors. Those in the survivorship phase control group had more comorbid conditions than those in the intervention group (mean 3 vs 2).

**Table 1:** Baseline and clinical characteristics by phase and group (N=160).

Characteristics		Treatment		Survivorship	
		Intervention (n=33)	Control (n=32)	Intervention (n=43)	Control (n=52)
Age (years), mean (SD)		64 (11)	64 (11)	64 (9)	63 (11)
**Sex,** **n (%)**					
	Male	18 (55)	19 (59)	3 (7)	4 (8)
	Female	15 (45)	13 (41)	40 (93)	48 (92)
**Cancer type,** **n (%)**					
	Prostate	18 (55)	18 (56)	0 (0)	0 (0)
	Breast	15 (46)	14 (44)	39 (91)	44 (85)
	Colorectal	0 (0)	0 (0)	4 (9)	8 (15)
**Multimorbidity^a^, n (%)**					
	Hypertension	11 (33)	12 (38)	19 (44)	20 (39)
	Depression or anxiety	13 (39)	8 (25)	7 (16)	11 (21)
	Chronic musculoskeletal	5 (15)	3 (9)	6 (14)	10 (19)
	Arthritis or rheumatoid arthritis	11 (33)	3 (9)	14 (33)	19 (37)
	Diabetes	3 (9)	5 (16)	5 (12)	12 (23)
	Thyroid disorder	5 (15)	3 (9)	7 (16)	14 (27)
	Hyperlipidemia	10 (30)	10 (31)	14 (33)	16 (31)
	Other^b^	27 (82)	22 (69)	33 (77)	42 (81)
Number of comorbid conditions*,* mean (SD)		3 (2)	2 (2)	2 (2)	3 (2)
**Racial or cultural group,** **n (%)**					
	White	30 (91)	29 (91)	40 (93)	47 (90)
	Other^c^	3 (9)	3 (9)	3 (7)	6 (12)
**Marital status,** **n (%)**					
	Married or living with a partner	25 (76)	24 (75)	31 (72)	39 (75)
	Other	8 (24)	8 (25)	12 (28)	13 (25)
**Education, n (%)**					
	Secondary school or less	7 (21)	11 (34)	12 (28)	15 (29)
	University or community college	16 (49)	8 (25)	16 (37)	24 (46)
	Bachelor’s degree or more	10 (30)	13 (41)	15 (35)	13 (25)
**Work status,** **n (%)**					
	Full-time, self-employed (≥30 hours per week)	10 (30)	10 (31)	13 (30)	15 (29)
	Retired	18 (55)	15 (47)	20 (47)	23 (44)
	Other	5 (15)	7 (22)	10 (23)	14 (27)

^a^Patients may appear in more than 1 category for multimorbidity.

^b^Other multimorbidities: patients with osteoporosis (n=11), asthma or chronic obstructive pulmonary disease or chronic bronchitis (n=15), cardiovascular disease (n=10), heart failure (n=5), stroke or transient ischemic attack (n=4), stomach problem (n=30), colon problem (n=9), cancer in the previous 5 years (n=1), kidney disease (n=1), chronic urinary problem (n=12), HIV (n=1), and other (n=25).

^c^Other racial or cultural groups: South Asian (n=1), Chinese (n=2), Black (n=2), Filipino (n=1), Arab (n=3), Southeast Asian (n=1), West Asian (n=1), and other (n=4).

### Study Outcomes

There were no statistically significant differences between the groups in change scores for the primary outcome of the TCC subscale (Table 2).

However, there were statistically significant group differences in the secondary measure of anxiety. In the treatment phase, there was a statistically significant difference between the groups in the change score for GAD-7 from baseline-to FUP1 (mean difference −2.3, 95% CI −4.3 to −0.3; *P*=.03). Similarly, in the survivorship phase, there was a statistically significant difference between the groups in the change score for GAD-7 between baseline and FUP1 (mean difference –1.7, 95% CI −3.2 to −0.1; *P*=.03) and between baseline and FUP2 (mean difference −2.4, 95% CI −4.0 to −0.8; *P*=.004). The baseline-to-FUP1 change score for PHQ-9 in the treatment phase approached statistical significance (mean difference −1.4, 95% CI −2.9 to 0.1; *P*=.06). There were no statistically significant group differences for patient experience (PPE-15).

In the GLM analyses of the NCQ TCC change score (Table 3), the group effects (intervention vs control) were not statistically significant in either the treatment or survivorship phase after adjustment for baseline variables.

In addition, separate GLM models were used for each secondary outcome. For secondary outcomes, there was a statistically significant improvement in the GAD-7 change score between baseline and FUP2 in the survivorship phase. The model accounted for 23% of the variation in the GAD-7 change score. The main effect for the intervention variable was statistically significant (*P*=.04; Multimedia Appendix 2). There were no significant findings for the secondary analyses examining the outcome change scores between baseline and FUP1.

**Table 2:** Change from baseline in outcome scores by instrument, phase, and group.

Instrument			Change from baseline to FUP1^a^									Change from baseline to FUP2^b^						
			Intervention, mean (SD)		Control, mean (SD)		Difference (SE)		*P* value		Intervention, mean (SD)			Control, mean (SD)		Difference (SE)		*P* value
**Treatment**																		
	NCQ^c^ TCC^d^	0.2 (0.6)		0.1 (0.5)		0.1 (0.1)		.57		0.5 (0.8)			0.2 (0.7)		0.3 (0.2)		.14	
	GAD-7^e^	−2.6 (4.4)		−0.4 (3.6)		−2.3 (1)		.03^f^		−2.4 (5.4)			−1.5 (4.6)		−1.0 (1.3)		.46	
	PHQ-9^g^	−0.4 (3)		1.0 (2.8)		−1.4 (0.7)		.06		0.5 (4.5)			0.7 (4.1)		−0.2 (1.1)		.85	
	PPE-15^h^	−0.3 (1)		−0.6 (2.0)		0.2 (0.9)		.80		−1.2 (1.6)			0.4 (2.4)		−1.6 (1.3)		.23	
**Survivorship**																		
	NCQ TCC	−0.2 (0.7)		−0.1 (0.6)		−0.2 (0.1)		.26		−0.2 (0.9)			−0.2 (0.6)		0.0 (0.2)		.86	
	GAD-7	−1.0 (4.4)		0.7 (2.9)		−1.7 (0.8)		.03^f^		−1.5 (4.7)			0.9 (3.1)		−2.4 (0.8)		.004^f^	
	PHQ-9	−0.2 (4)		0.0 (2.9)		−0.2 (0.7)		.80		−1.1 (3.3)			−0.1 (4.0)		−0.9 (0.8)		.22	
	PPE-15	0.5 (1.1)		−0.1 (3.1)		0.6 (1)		.55		−0.2 (2.8)			−1.5 (1.9)		1.2 (1)		.21	

^a^FUP1: first follow-up.

^b^FUP2: second follow-up.

^c^NCQ: Nijmegen Continuity Questionnaire.

^d^TCC: team and cross-boundary continuity.

^e^GAD-7: Generalized Anxiety Disorder Screener.

^f^*P*≤.05 (*t* tests were used to compare change scores).

^g^PHQ-9: Patient Health Questionnaire on Major Depression.

^h^PPE-15: Picker Patient Experience Questionnaire.

**Table 3:** General Linear Model analysis results for Nijmegen Continuity Questionnaire (NCQ) team and cross-boundary continuity (TCC) baseline-to-second follow-up change score.

Model term			Coefficient		95% CI		*P* value
**Treatment**							
	Intercept	1.1		−0.9 to 3.1		.26	
	Group (intervention vs control)	0.1		−0.4 to 0.6		.66	
	Cancer type (breast vs prostate)	−0.2		−0.7 to 0.4		.59	
	Age (continuous)	0		0 to 0		.52	
	Total comorbid conditions (continuous)	0		−0.1 to 0.1		.55	
	Baseline NCQ TCC	−0.6		−1.1 to −0.2		.01	
	Baseline NCQ PC1^a^	0.1		−0.2 to 0.4		.41	
	Baseline NCQ PC2^b^	0.3		0 to 0.7		.07	
	Intervention × cancer type (interaction)	0.3		−0.5 to 1.1		.49	
**Survivorship**							
	Intercept	2.2		0.5 to 4		.01	
	Group (intervention vs control)	0		−0.3 to 0.3		.89	
	Sex (female vs male)	−1		−1.8 to −0.2		.02	
	Cancer type (breast vs colorectal)	0.5		−0.1 to 1.2		.13	
	Age (continuous)	0		0 to 0.1		.21	
	Total comorbid conditions (continuous)	0.1		0 to 0.1		.14	
	Baseline NCQ TCC	−0.6		−0.9 to −0.3		<.001	
	Baseline NCQ PC1	0		−0.2 to 0.2		.87	
	Baseline NCQ PC2	0.2		−0.1 to 0.4		.20	

^a^PC1: personal continuity relating to the PCP.

^b^PC2: personal continuity relating to the cancer specialist.

### Health Care Use

Most patients in both the treatment and survivorship phases had not seen their PCP in more than 4 weeks across baseline (46/65,71% in treatment; 69/95, 73% in survivorship), FUP1 (48/65, 74% in treatment; 56/95, 59% in survivorship), and FUP2 (41/65, 63% in treatment; 58/95, 61% in survivorship; Table 4). Although for most patients in the survivorship phase, the reason for their last PCP visit was for another medical concern or general checkup, patients in the treatment phase indicated that their last visit to their PCP was usually for a cancer-related issue (ie, to discuss cancer-related symptoms or treatment).

**Table 4:** Health care use by phase, assessment, and group.

Resource				Baseline				FUP1^a^				FUP2^b^									
				Intervention		Control		Intervention		Control		Intervention		Control							
**Treatment**																					
	**Last seen PCP^c^, n (%)**																				
		1-2 weeks ago	7 (21)		3 (9)		5 (15)		4 (13)		3 (9)		8 (25)								
		3-4 weeks ago	5 (15)		4 (13)		7 (21)		1 (3)		5 (15)		3 (9)								
		>4 weeks ago^d,e,f^	21 (64)		25 (78)		21 (64)		27 (84)		20 (61)		21 (66)								
		Missing	0 (0)		0 (0)		0 (0)		0 (0)		5 (15)		0 (0)								
**Reason for last visit to the PCP, n (%)**																					
		General checkup	6 (18)		9 (28)		6 (18)		8 (25)		7 (21)		9 (28)								
		Discuss symptoms that may be related to my cancer	5 (15)		6 (19)		2 (6)		2 (6)		5 (15)		1 (3)								
		Discuss cancer treatment	7 (21)		3 (9)		6 (18)		6 (19)		7 (21)		7 (22)								
		Discuss another medical concern	8 (24)		9 (28)		12 (36)		8 (25)		7 (21)		10 (31)								
		Other	13 (39)		9 (28)		12 (36)		11 (34)		12 (36)		9 (28)								
**Survivorship**																					
	**Last seen PCP,** **n (%)**																				
		1-2 weeks ago	9 (21)		7 (14)		7 (16)		12 (23)		6 (14)		6 (12)								
		3-4 weeks ago	8 (19)		2 (4)		8 (19)		8 (15)		10 (23)		12 (23)								
		>4 weeks ago^d^	26 (60)		43 (83)		28 (65)		28 (54)		27 (63)		31 (60)								
		Missing	0 (0)		0 (0)		0 (0)		4 (8)		0 (0)		3 (6)								
	**Reason for last visit to the PCP,** **n (%)**																				
		General checkup	10 (23)		14 (27)		11 (26)		17 (33)		18 (42)		24 (46)								
		Discuss symptoms that may be related to my cancer	5 (12)		6 (12)		1 (2)		1 (2)		5 (12)		1 (2)								
		Discuss cancer survivorship	2 (5)		3 (6)		8 (19)		5 (10)		1 (2)		3 (6)								
		Discuss another medical concern	19 (44)		23 (44)		18 (42)		22 (42)		12 (28)		14 (27)								
		Other	11 (26)		11 (21)		15 (35)		13 (25)		12 (28)		13 (25)								

^a^FUP1: first follow-up.

^b^FUP2: second follow-up.

^c^PCP: primary care provider.

^d^The baseline questionnaire included more response options than the follow-up questionnaire (1-3 months ago, 4-6 months ago, and >6 months ago), which had collapsed into the >4 weeks ago category.

^e^At baseline in the intervention group for the treatment phase, 12 patients (36%) indicated 1 to 3 months ago, 5 patients (15%) indicated 4 to 6 months ago, and 4 patients (12%) indicated more than 6 months ago. At baseline in the control group for the treatment phase, 18 patients (56%) indicated 1 to 3 months ago, 5 patients (16%) indicated 4 to 6 months ago, and 2 patients (6%) indicated more than 6 months ago.

^f^At baseline in the intervention group for the survivorship phase, 12 patients (28%) indicated 1 to 3 months ago, 10 patients (23%) indicated 4 to 6 months ago, and 4 patients (9%) indicated more than 6 months ago. At baseline in the control group for the survivorship phase, 26 patients (50%) indicated 1 to 3 months ago, 13 patients (25%) indicated 4 to 6 months ago, and 4 patients (8%) indicated more than 6 months ago.

### Hospital EMR Data

In total, there was minimal communication from PCPs to specialists recorded in the EMR for both the intervention and control groups. There was 1 patient for whom a PCP called or sent a fax 3 times in the treatment phase control group and 4 patients (1 intervention and 3 control) for whom a PCP called or sent a fax once in the survivorship phase (Table 5). The main reason for PCP calls and faxes was for symptom management.

There were more calls from patients in the treatment phase (61/69, 88%) than in the survivorship phase (8/69, 12%). In the treatment phase, 56% (34/61) and 44% (27/61) calls were logged in the intervention and control groups, respectively. All the patient calls logged in the survivorship phase were in the control group. The primary reasons for patient calls were symptom management or other reasons.

**Table 5:** Data abstracted from hospital electronic medical record (N=160).

Characteristic			Treatment				Survivorship		
			Intervention (n=33)		Control (n=32)		Intervention (n=43)		Control (n=52)
**Calls and faxes from PCP^a^, n (%)**									
	0	33 (100)		31 (97)		42 (98)		49 (94)	
	1	0 (0)		0 (0)		1 (2)		3 (6)	
	≥2	0 (0)		1 (3)		0 (0)		0 (0)	
**Reason for calls and faxes from PCP,** **n (%)**									
	Treatment	0 (0)		0 (0)		0 (0)		1 (33)	
	Symptom management	0 (0)		2 (67)		1 (100)		2 (67)	
	Other	0 (0)		1 (33)		0 (0)		0 (0)	
**Calls from patient,** **n (%)**									
	0	20 (61)		18 (56)		43 (100)		47 (90)	
	1	3 (9)		6 (19)		0 (0)		3 (6)	
	2	3 (9)		4 (13)		0 (0)		1 (2)	
	≥3	7 (21)		4 (13)		0 (0)		1 (2)	
**Reasons for calls from patient,** **n (%)**									
	Management of comorbid conditions	2 (6)		1 (4)		0 (0)		0 (0)	
	Symptom management	22 (65)		13 (48)		0 (0)		2 (25)	
	Treatment	2 (6)		1 (4)		0 (0)		0 (0)	
	Other	8 (24)		12 (44)		0 (0)		6 (75)	

^a^PCP: primary care provider.

## Discussion

### Principal Findings

This study examined the effect of a communication system for PCPs and cancer specialists on patient-perceived continuity of care, anxiety, depression, and patient experience of their health care. Although the results of this trial did not show an intervention effect on patient-perceived continuity of care as measured by the NCQ TCC, there were significant improvements in anxiety among patients in the intervention group compared with the control group, as measured by the GAD-7.

Previous research has suggested that the NCQ is a reliable and valid measure, with no ceiling effects observed for the TCC subscale in a study involving patients with pancreatic cancer [38]. The NCQ TCC results may have been affected by too much noise (sources of variation) owing to the high volume of health care activities patients are involved in (particularly during the treatment phase), and it may have been difficult for the intervention to have an effect. In the treatment phase, we also were unable to reach our target sample size, resulting in a smaller sample. Although the NCQ TCC items were appropriate for examining patient perceptions of communication and sharing information between their PCP and cancer specialists, many patients responded, “I do not know,” which we recoded into “neutral” for the primary analysis. However, there were no significant findings for the primary outcome with recoding, sensitivity analysis, or secondary analysis examining the NCQ TCC change scores between baseline and FUP1. Similar to this study, Hopstaken et al [38] reported that 36% to 41% of patients did not know how to respond to the TCC subscale items in their study involving patients with pancreatic cancer. We learned that most patients were unaware of the communication between their health care providers regarding their care and may have assumed that it was adequate unless specific problems arose (data not shown). This raises the question about the best measures and methods to examine patient perceptions of continuity of cancer care and highlights the strengths of using a mixed methods approach to explain and support trial findings with qualitative data [39].

Patients living with cancer are more likely to have anxiety than the general population, although rates may vary based on the disease site, differences in prognosis, and side effects [40]. Anxiety tends to be a comorbid condition with depression, with up to two-thirds of patients with depression also having symptoms of clinical anxiety [40]. Although on average, patient anxiety scores in this study were mild (5 is the cutoff for mild anxiety, whereas 10 is considered moderate anxiety [41]), findings indicated a statistically significant difference in GAD-7 change scores between baseline and FUP1 and baseline and FUP2 during the survivorship phase. The results of the GLM analysis supported these findings. There was also a significant difference in anxiety change scores between baseline and FUP1 during the treatment phase, although this was not sustained to FUP2. We did not find significant results for the secondary outcomes of depression and patient experience of their health care. Although it is encouraging that an intervention effect was observed for anxiety, previous intervention studies focusing on improving cancer care coordination or continuity of care did not demonstrate an influence on anxiety or depression [42-44]. The lack of positive findings for patient experience of their health care may have been influenced by missing data, as patients were asked to answer questions based on contact with health care providers in the past month (if they did not have an appointment in the past month, patients did not answer those items). When asked about the last time they visited their PCP, most patients in both the treatment and survivorship phases indicated that their visit was >4 weeks before the assessment (which may further explain the lack of significant findings for the primary outcome).

Our secondary research question regarding the influence of eOncoNote on communication between PCPs and cancer specialists was answered by analyzing hospital EMR data. The results from the hospital EMR data abstraction suggested that there were few calls made by PCPs or faxes sent to the cancer center during the treatment and survivorship phases in both groups. Other studies have reported infrequent communication between oncologists and PCPs [45] and have noted that limited communication was a barrier to the integration of PCPs into the care of cancer survivors [46]. If patients were not seeing their PCP during active treatment, PCPs may not have had a reason to contact the cancer center. During the survivorship phase, we collected data for 1 year following the patient’s discharge to their PCP. It is possible that more PCP contacts to the cancer center occur later in the patient’s cancer trajectory, as they complete follow-up diagnostic imaging (eg, annual mammogram) or other cancer surveillance tests. It is also possible that the use of the eOncoNote system reduced the need for calls between the PCPs and specialists in the intervention group. Perhaps, there was less need for calls between PCPs and specialists overall for this well-followed patient population.

### Strengths

This study builds on previous research conducted by our team as part of the CanIMPACT program of research. Tomasone et al [42] conducted a systematic review of interventions designed to improve coordination between primary care and cancer specialists. Of the 22 publications included in the systematic review, none of the intervention studies provided a specific tool for facilitating 2-way communication between PCPs and cancer specialists. Furthermore, an environmental scan conducted by our team [47], which examined Canadian initiatives designed to improve cancer care coordination, found that most focused on posttreatment follow-up care. The primary strategy reported in the environmental scan was nurse or patient navigation, whereby patient navigators liaise with patients (providing education, support, and connection with community resources), the cancer system, and PCPs. This study addressed the gaps identified in previous research by testing a system (eOncoNote) specifically designed to facilitate one-on-one communication between PCPs and cancer specialists and examining the application of the system while patients received active treatment and during posttreatment follow-up care.

### Limitations

We encountered several challenges and limitations. First, we did not reach our patient recruitment target in the treatment phase. Over the course of patient recruitment, it became increasingly difficult to recruit new participants, as PCPs could only have 1 patient participating in the trial to minimize the potential for contamination between study groups (ie, if a patient was interested in participating and their PCP already had another patient in the trial, they were ineligible to enroll). In addition, only PCPs who had already signed up for eConsult could participate, thus further reducing the number of potential patients that could be enrolled. The COVID-19 pandemic caused further difficulties in patient recruitment.

There was no mechanism to ensure that patients were blinded to their group allocation; however, we learned that most patients were unaware of the communication between their health care providers, suggesting that they likely did not know whether they were randomized to the intervention or control group.

Within the context of the survivorship phase, the eOncoNote discussion lasted for 1 year after the patient was discharged to their PCP. Patients may experience late effects beyond that period, and questions may arise when patients visit their PCP (clinical guidelines recommend that the frequency of visits be adjusted to the individual needs of breast cancer survivors [48] and every 6 months within the first 5 years of follow-up for colorectal cancer survivors [49]). Considering the timeline restrictions for this project, we could not examine the long-term outcomes (such as the impact of eOncoNote on collaborative relationships between PCPs and cancer specialists), and we were unable to include patient caregivers as participants in this study.

Some limitations were specific to the eOncoNote system setup. The eOncoNote system did not support EMR integration, which may have impacted uptake among health care providers. Although ideally, the discussion between health care providers would follow a patient across the cancer care continuum, we were unable to examine communication longitudinally because of the current functionality of eOncoNote. The eOncoNote system was not designed to be a managed service, which would require a case manager to support a longitudinal discussion and was beyond the scope of this study.

### Conclusions

The results of this trial demonstrate that health care provider access to a web-based asynchronous communication system did not impact patient perceptions regarding continuity of care but may have been a factor in reducing cancer survivors’ anxiety after discharge to primary care. Future research could examine the applicability of the eOncoNote system for different cancer disease sites, other phases of cancer care, and more complex patients and explore the possibility of adapting the system to facilitate longitudinal communication across a patient’s cancer journey.

## Appendix

Multimedia Appendix 1 CONSORT (Consolidated Standards of Reporting Trials) diagram.

Multimedia Appendix 2 General Linear Model for Generalized Anxiety Disorder Screener baseline-to-second follow-up change score in the survivorship phase.

Multimedia Appendix 3 CONSORT-eHEALTH checklist (V 1.6.1).

## Abbreviations

BASE: Building Access to Specialists through eConsultation
CanIMPACT: Canadian Team to Improve Community-Based Cancer Care along the Continuum
CONSORT: Consolidated Standards of Reporting Trials
EMR: electronic medical record
FUP1: first follow-up questionnaires, administered at 1 month (treatment phase) or 6 months (survivorship phase)
FUP2: second follow-up questionnaires, administered at 4 months or 6 months (for patients receiving treatment for prostate and breast cancer, respectively), and 12 months (for patients in the survivorship phase)
GAD-7: Generalized Anxiety Disorder Screener
GLM: General Linear Model
NCQ: Nijmegen Continuity Questionnaire
PCP: primary care provider
PHQ-9: Patient Health Questionnaire on Major Depression
PPE-15: Picker Patient Experience Questionnaire
RA: research assistant
TCC: team and cross-boundary continuity
