# Supplementary figures and images for: Web-Based Asynchronous Tool to Facilitate Communication Between Primary Care Providers and Cancer Specialists: Pragmatic Randomized Controlled Trial
Source: J Med Internet Res. 2023 Jan 18;25:e40725. doi: 10.2196/40725 (PMC9892983; doi:10.2196/40725)

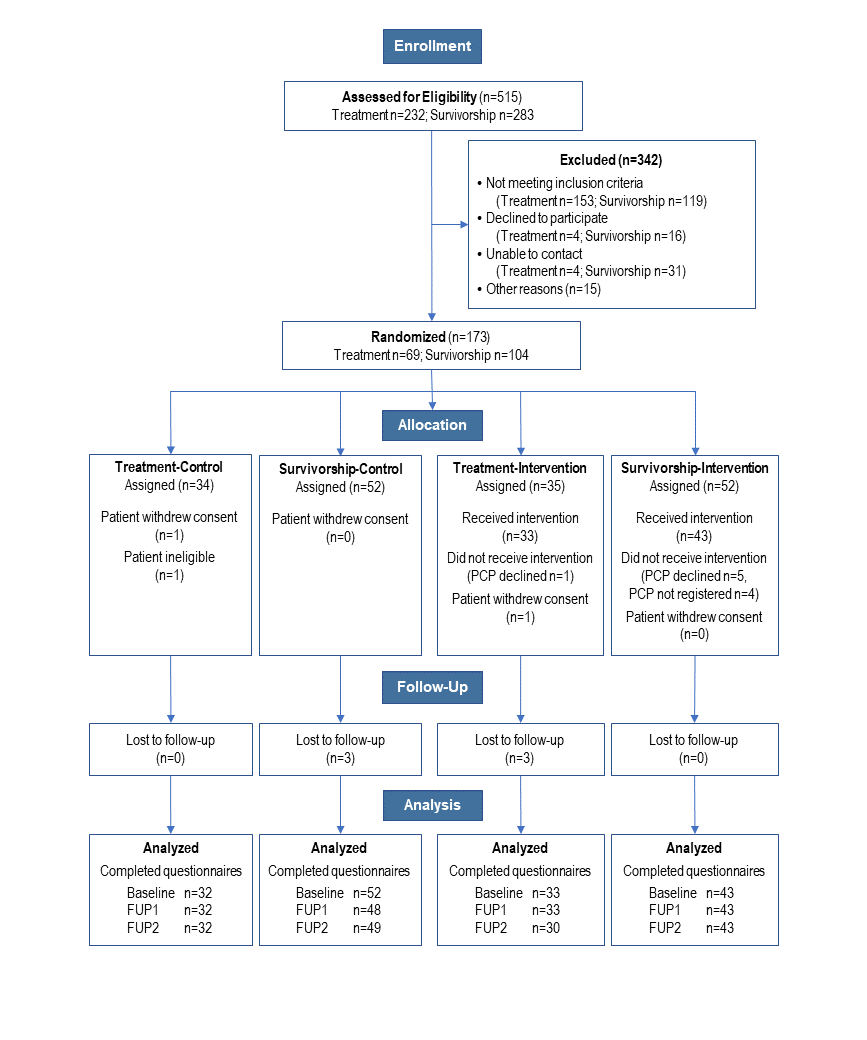

Supplement: Multimedia Appendix 1 [file jmir_v25i1e40725_app1.png]
