# Supplementary material for: Web-Based Asynchronous Tool to Facilitate Communication Between Primary Care Providers and Cancer Specialists: Pragmatic Randomized Controlled Trial
Source: J Med Internet Res. 2023 Jan 18;25:e40725. doi: 10.2196/40725 (PMC9892983; doi:10.2196/40725)
Supplement: Multimedia Appendix 2 [file jmir_v25i1e40725_app2.docx]

## Multimedia Appendix 2: GLM for GAD-7 Baseline-to-FUP2 Change Score in the Survivorship Phase

| Factor | Coefficient | 95% CI | | P-value |
| --- | --- | --- | --- | --- |
|  |  |  |  |  |
| Intercept | 1.08 | -4.97 | to 7.13 | .72 |
| Group (intervention vs control) | -1.60 | -3.14 | to -.06 | .04 |
| Sex (female vs male) | 5.36 | 1.09 | to 9.63 | .01 |
| Cancer type (breast vs colorectal) | -4.55 | -7.94 | to -1.17 | .01 |
| Age (continuous) | -.02 | -.11 | to .06 | .63 |
| Total comorbid conditions (continuous) | .37 | -.06 | to .79 | .09 |
| Baseline GAD-7 | -.38 | -.57 | to -.19 | <.001 |
